# High clutch failure rate due to unpredictable rainfall for an ephemeral pool-breeding frog

**DOI:** 10.1007/s00442-022-05139-2

**Published:** 2022-03-05

**Authors:** John Gould, John Clulow, Simon Clulow

**Affiliations:** 1 Conservation Biology Research Group, School of Environmental and Life Sciences, University of Newcastle, University Drive, Callaghan, NSW 2308 Australia; 2 Centre for Conservation Ecology and Genomics, Institute for Applied Ecology, University of Canberra, Bruce, ACT 2617 Australia

**Keywords:** Anuran, Offspring survival, Rainfall intermittency, Reproduction, Variable environment

## Abstract

Animals that reproduce in temporary aquatic systems expose their offspring to a heightened risk of desiccation, as they must race to complete development and escape before water levels recede. Adults must therefore synchronise reproduction with the changing availability of water, yet the conditions they experience to trigger such an event may not relate to those offspring face throughout development, potentially leading to clutch failure. The sandpaper frog (*Lechriodus fletcheri*) breeds in ephemeral pools that dry within days to weeks after rainfall has ceased. We examined whether spawning frequency and offspring survival differed across two consecutive breeding seasons based on (1) rainfall at the moment of oviposition and throughout offspring development, and (2) pool volume, given their combined effect on hydroperiod. Reproduction was triggered by rainfall, with more spawn laid during periods of greater rainfall and in larger pools. While pool size was a predictor of offspring survival, rainfall during oviposition was not. Rather, follow-up rain events were required to prevent pools drying prior to metamorphosis, with rainfall evenness during development the strongest predictor of reproductive success. High clutch failure rates recorded in both seasons suggest that adults do not have the capability to predict rainfall frequency post-oviposition. We thus conclude that unpredictable rainfall leading to premature desiccation of spawning sites is the primary source of pre-metamorphic mortality for this species. Understanding the influence of rainfall predictability on offspring survival could be critical in predicting the effects of altered hydroperiod regimes due to climate change for species that exploit temporary waters.

**Supplementary Information:**

The online version contains supplementary material available at 10.1007/s00442-022-05139-2.

## Introduction

Many animals reproduce in temporary aquatic habitats, such as ephemeral pools and streams that only become available in the landscape periodically and often for short periods of time (Heyer et al. 1975; Wilbur 1980; Williams 2006; Colburn et al. 2008; Furness et al. 2015). This can confer an offspring survival advantage by reducing exposure to predators confined to more permanent systems (Duellman and Trueb 1986; Skelly 1996; Wellborn et al. 1996), yet expose individuals to an increased risk of total clutch failure as the rate of system desiccation may exceed the minimal developmental time required before offspring can escape (Denver et al. 1998; Saward-Arav et al. 2016). Adults must be able to perceive and rapidly respond to the recharging of temporary aquatic systems. Their decision making in terms of the timing and location of reproduction, thus, plays a critical role in determining the chances of their offspring surviving. Yet, the conditions that are triggers for reproduction may not necessarily correlate with the conditions offspring are subsequently exposed to throughout development.

In highly ephemeral water bodies, hydroperiod length is associated with rainfall quantity and evenness, as both dictate how much water makes it into a system over time (Newman 1989; Zacharias et al. 2007). A large rain event can fill a water body up to and beyond its maximum water holding capacity, though a single recharge may not be sufficient to maintain water levels over the course of offspring development once rainfall has ceased, particularly in small systems that have smaller water holding capacities and lack groundwater connections (Brunner et al. 2011). Instead, the intermittency of rainfall may play a stronger role in determining how regularly water levels are topped up and how often these systems revert back to their dry phase (Batzer and Boix 2016). The length of time over which temporary aquatic systems retain water and, thus, their rate of disturbance is also dependent on qualities such as volume, surface area, depth and sediment lining, which dictate their innate water retention capacity by influencing rates of water loss through seepage and evaporation (Chang et al. 1974; Black 1976; Fredlund et al. 1994; Schneider and Frost 1996). At its most fundamental level, it is this interplay between water retention (waterbody structure) and water inundation (rainfall) that determines the level of both temporal and spatial variability in hydroperiod among potential oviposition sites.

While breeding by amphibians is commonly linked with rainfall (Duellman and Lizana 1994), it is particularly associated with reproductive success for species that use ephemeral waters, given the habitat disturbance that arises when these systems begin to dry once rainfall has ceased (Werner and McPeek 1994; Skelly et al. 1999). If rainfall is variable but predictable, phenotypic plasticity in the timing of reproduction improves the chances of reproduction being synchronised with the recharging of these systems (Blair 1960; Nager and van Noordwijk 1995). This is typically observed in temperate amphibians, which restrict breeding to a particular season as an adaptation to annual variation in environmental conditions, including rainfall (Duellman and Trueb 1986; Feder and Burggren 1992). Though seasonal breeding may reduce the risk of offspring mortality due to desiccation, it is possible that rainfall intensity, duration, and frequency are still variable, causing conditions to vary between those that are optimal and sub-optimal for offspring survival across a season. If oscillations in rainfall patterns are unpredictable, there may be insufficient cues to synchronise reproduction with the onset of sustained rainfall that is required to keep ephemeral systems from drying out prematurely. The difficulty in assessing when to oviposit is further confounded by the choice of where to oviposit if sites within the landscape dry at variable rates (Gómez-Rodríguez et al. 2009), and adults are not able to predict likely hydroperiods based on immediate waterbody attributes (e.g. waterbody size) (Goldberg et al. 2006).

Such unpredictability in hydroperiods can result in a level of uncertainty in reproductive success that should be evident in a population through a high rate of clutch failure. Although direct relationships between offspring survival and breeding site ephemerality have been established in various species (Licht 1974; Shoop 1974; Rowe and Dunson 1995), these remain difficult to assess under natural conditions due to the difficulties in following offspring through development and the impracticality of assessing survival in water bodies that harbour multiple egg clutches from different parents. Instead of measuring the proportion of offspring from each clutch that survive, a more practical measure of survival is the proportion of clutches of a species in a region that are laid in water bodies that have sufficient hydroperiods for successful metamorphosis to be achievable. Using this approach, reproductive success is measured at the ‘clutch’ level based on the presence of offspring in waterbodies beyond the minimum development period required to reach metamorphosis, thereby providing an estimate of offspring survival rates within a population. This technique allows assessment of the impact of rainfall variability over the breeding season on reproduction.

The sandpaper frog, *Lechriodus fletcheri*, is an Australian anuran that breeds during intermittent rainfall periods over an extended breeding season (September–February), and exclusively in small, highly ephemeral pools. These pools often dry within a matter of days or weeks after rainfall has ceased given their limited water holding capacity, often resulting in tadpoles failing to complete development (*Pers. Obs.*). We used data from two consecutive *L. fletcheri* breeding seasons to determine whether adults respond adaptively to rainfall, with the fitness benefits of this behaviour assessed in terms of offspring survival until metamorphosis. We examined whether spawning frequency and clutch success differed across both seasons based on (1) rainfall at the moment of oviposition and then later throughout offspring development, and (2) pool size, given the effect of each on hydroperiod. We hypothesised that the amount and distribution of follow-up rainfall that occurred over the period encompassing offspring development would influence survival more than rainfall at the moment spawning occurred. This is because run-off will occur once pools have reached water holding capacity and drying out events will occur if water levels are not regularly topped up by additional rainfall given the ephemerality of breeding sites. We expected clutch success to be generally low across the season due to the intermittency and unpredictability of rainfall, unless spawning was correlated non-randomly with periods of follow-up rainfall post-spawning, which would be evidence of the capacity of the species to anticipate these future events. We also examined historical rainfall data over the past century to assess whether rainfall patterns within this species’ range are changing as a result of climate change. Understanding how species are impacted by rainfall variability is required to assist in predicting their sensitivity to future changes in rainfall patterns, as it is reasonable to expect the reproductive success of species restricted to breeding in temporary systems to be disproportionately impacted by climate change (Brooks 2009; Abney et al. 2019).

## Materials and methods

### Field site

**Fig. 1:**
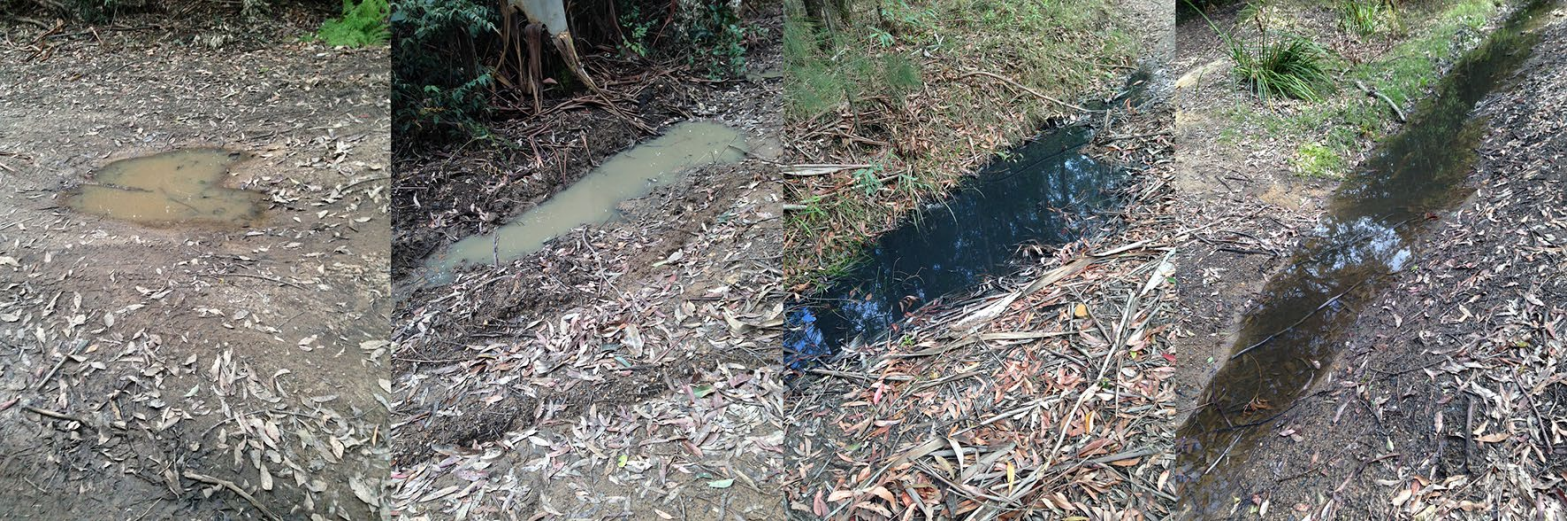
*Lechriodus fletcheri* breeding pools surveyed within the Watagan Mountains. Pools were photographed after rainfall and were full

The study was conducted in the Watagan Mountains, NSW, Australia (approximately 33° 00′ 30.6 S, 151° 23′ 15.7 E) during the 2015/16 and 2016/17 breeding seasons. This is one of several key areas along Australia’s east coast where this species is found (Anstis 2017). Potential breeding pools within the study area were surveyed during periods of rainfall for the presence of *L. fletcheri* egg clutches (spawns). We grouped consecutive days of rainfall into ‘rain events’, defined as all days of rainfall above 0.2 mm separated by < 24 h. A rain event was deemed to be over if more than 24 h passed with rainfall below 0.2 mm, at which point the next day of rainfall above 0.2 mm signalled the start of a new rain event. We located 84 pools in the 2015/16 breeding season and 71 pools in the 2016/17 breeding season, by driving or walking along fire trails and roads extending throughout the study site during initial rainfall in each season. Pools were located on the forest floor and generally lacked submerged or surrounding vegetation, except for fallen leaves (Fig. 1). These pools were rarely used by sympatric species, except for two pools that were also periodically used by *Litoria chloris* and *Pseudophryne coriacea*. It is likely that additional pools were available within the study site which were not surveyed, while three large, permanent ponds present within the site were never used and not included in our analyses given that *L. fletcheri* is an ephemeral pool breeder. Pools were surveyed over 52 and 59 nights across the span of the 2015/16 and 2016/17 breeding seasons, respectively. Nearly half of all surveys occurred on nights outside of rain events, with the mean interval between survey nights being 2.6 days (SD = 2.9).

### Pool surveys

At the beginning of each season, the maximum volume of each pool was calculated based on surface area and average depth measurements. Pool surface area was determined by measuring the surface length and width or diameter, depending on pool geometric form, while average depth was determined by measuring three points along the vertical and horizontal axis of each pool, including at the centre and at ¾ distances from the edge. Maximum volume was used as a measure of pool size in all subsequent analyses.

Across each season, we recorded the total number of days each pool contained water. We also recorded the length of each hydroperiod, defined as the total number of consecutive days in which a pool contained freestanding water between filling and drying. These measures were calculated based on the known presence of water in pools during survey events. The presence of water on days between survey events was imputed based on data collected during surveys. In particular, if pools were wet on a survey event, they were deemed to have continued to remain wet until the next survey event. If pools were dry on a survey event, they were deemed to have remained dry until the next survey event in which water was found to be present, conditional on there being no rain events over this interval.

### Spawn surveys

On each survey night, pools were inspected for the presence of *L. fletcheri* spawns. Individual spawns were aged to the day they were oviposited based on the developmental stage of the residing embryos (Gosner 1960). In most cases, individual spawns were recorded within a day of being oviposited, though some were initially found up to 2–4 days post-oviposition depending on when survey events occurred. As multiple *L. fletcheri* spawns were often laid within pools over several days, they were distinguished from each other by the placement of a thin strip of coloured paper on their top surface.

Pools were surveyed regularly to determine whether offspring from each spawn remained alive, which was apparent by the lack of disintegration of the embryos, their continued developmental progression and movements at later development stages. All spawns were found to contain live embryos over the 3–4 days prior to hatching (Gould et al. 2021b), even when stranded in completely desiccated pools, due to the moist environment created by the frothed oviduct fluid making up the matrix of the spawn body. However, spawns that were not in water at the time of tadpole hatching were scored as giving rise to no surviving offspring (clutch failure), as there was no means by which tadpoles could survive in the absence of water. If hatching occurred while spawns were immersed in water, pools were repeatedly surveyed for the presence of the tadpoles by visual inspection of the water and dip-netting of the mid-section of the pool. The period of time living tadpoles continued to be observed was used to determine the maximum potential survival period of offspring from each spawn. Tadpoles were deemed to have perished if they were not seen in pools for two consecutive survey nights or if the pool in which they were developing dried completely. If clutch failure did occur, the cause of failure was recorded, including whether it was due to declining water levels or complete pool desiccation. Egg hatching in this species is considered to take place 3–4 days post-oviposition in the field, while a range of combined egg and tadpole developmental times to metamorphosis (23–32 days) have been suggested by Anstis (2017) for spawn raised under laboratory conditions. It is likely that some offspring in the field are able to complete development more rapidly than expected under favourable conditions, given that developmental plasticity in response to changing water levels, food resources and temperature is likely for tadpoles of this species. We considered spawns to have given rise to embryos that successfully reached metamorphosis if at least one tadpole was present after a critical time threshold where escape from pools would subsequently be possible. To account for the possibility of tadpole developmental plasticity, we compared survival rates using two different time thresholds: an extreme scenario where tadpole development took only 2 weeks (total time between oviposition and metamorphosis being 18 days), and a less extreme scenario where tadpole development took 3 weeks (total time between oviposition and metamorphosis being 25 days). This allowed us to determine the minimum and maximum number of spawns laid each season that potentially gave rise to offspring that survived to a point where metamorphosis could theoretically be completed (beyond which their survival would not be dependent on pool hydroperiod), and to thus define the rate of reproductive success of adults in this population.

On some occasions, multiple spawns were oviposited in the same pool during a rain event, preventing hatched tadpoles from being traced back to a particular spawn of origin. Under these circumstances, all spawns were deemed to have tadpoles still present in the pool as long as at least one tadpole continued to be located in that system up to the threshold periods, potentially leading to an overestimation of clutch success. On some occasions, older tadpoles from spawns oviposited in a previous rain event were present in a pool alongside the newly hatched tadpoles of recently oviposited spawns. This occurred when pools retained standing water between periods of rain. These two different cohorts could be easily distinguished as the rapid developmental rate of tadpoles in this species resulted in a clear delineation in size between individuals produced in different rain events.

### Analysis of pool wetness

The effect of pool size on hydroperiod length was analysed using linear mixed effect modelling. We only analysed those pools which were used for spawning at least once in either breeding season. Season and pool were included as random effects. Rainfall data were not included in this analysis, given that all pools within the study area were exposed to the same rainfall conditions.

### Analysis of spawning activity

It was clear from our field observations that spawning was triggered in response to rainfall, and that some pools surveyed were never used for spawning. Thus, we were interested in determining the temporal and spatial distribution of spawning activity across the breeding season. We focused in on those pool nights where spawning occurred at least once in either season. The effect of rainfall on the temporal distribution of spawning (model 1), and pool size on the spatial distribution of spawning (model 2) were analysed using separate generalised linear mixed effects models with a Poisson distribution and log link function. In model 1, the predictor variable was the cumulative total amount of rainfall that fell 2 days prior, during and 2 days after the date of oviposition for each spawn. It was important to include rainfall on the day of spawning and the few days following, to account for the capacity for adults to rapidly respond to and anticipate the start of a rainfall event. In model 2, the predictor variable was the size of each pool measured in terms of log maximum volume. The response variable in both models was the count of total spawns oviposited per pool or per date, with season included as a random effect in both models. The effects of pool size and rainfall were analysed in separate models, as it was assumed that all pools and days of rainfall were available for spawning for all adults in the population, and as we wanted to assess the overall effect across the entire season. Rainfall data were obtained from the Bureau of Meteorology (www.bom.gov.au, 2018) for the closest weather station (Cooranbong ID: 061,412) located approximately 8–10 km from the study site.

### Analysis of clutch success

The proportion of spawns with offspring that survived beyond the set time thresholds for metamorphosis (18 or 25 days) were calculated for field data obtained from both seasons. However, we decided to perform subsequent analyses on survival based on the less rapid tadpole developmental rate, as we expected most offspring to metamorphose after this period of time. As with spawning activity, we were interested in determining the temporal and spatial distribution of spawning success across the breeding season. The effect of (i) 5 days rainfall around the oviposition period and (ii) pool size on the chances of subsequent clutch success were analysed using a generalised linear mixed effects model with a binomial distribution and link function. In this model, counts of the number of spawn that did/did not give rise to offspring surviving to metamorphosis were included as a binary response variable using the cbind function in R. Pool-date was included as a random effect to account for differences in the total number of successful and failed spawn oviposited per pool per date; spawn laid on the periphery of pools occasionally dried and failed due to receding water levels while those laid in the centre survived.

Beyond simply the total amount of rain that fell at the start of oviposition, we hypothesised that the amount and consistency of subsequent rainfall over the developmental period may have a greater influence on offspring survival, given the extreme ephemerality of breeding pools which would often dry repeatedly across the season. In light of this, we analysed the effect of rainfall over the developmental period on offspring survival using Cox proportional-hazard regression modelling (Cox 1972). Offspring survival was modelled from the day offspring were oviposited in spawns (*t*_0_) to the day of failure up until 25 days, after which tadpoles were classified as surviving to metamorphosis and recorded as censored data as we considered escape from pools possible after metamorphosis had occurred. We examined the effect of rainfall distribution in terms of rainfall evenness, as well as rainfall amount in terms of cumulative rainfall. Rainfall evenness was calculated using the Shannon diversity index in combination with Pielou’s formula (Pielou 1966) (note that on days with no rainfall, a small value must be added as this index does not take into consideration ‘0’ values). Pool size was also included as a predictor, while season and spawn identification was included as random effects.

As Cox models estimate a hazard function based on all subjects alive on a given day, both rainfall predictors were considered time dependent and assessed in terms of their cumulative values analysed from *t*_0_. Although multiple spawns were often laid in pools on the same day, we only analysed each pool–date combination once based on the spawn with the longest survival length. Hazard ratios were subsequently calculated for each predictor to determine their relative effect on offspring survival in each season, with values > 1 indicative of variables that increase the risk of death and values < 1 indicative of variables with a protective effect.

### Historical seasonal rainfall

We examined historical rainfall data to determine whether rainfall patterns have changed over time. Rainfall patterns at the study site for the *L. fletcheri* breeding season (September to February) were examined from 1906 to 2020, with data obtained from the closest weather station (BOM ID: 061,012) 8–10 km away from the study site. Missing data for the period 2011–2015 were obtained from a secondary weather station (BOM ID: 061,412) that was also 8–10 km away from the study site. Missing data (< 0.1% of days) prior to 2011 were given zero rainfall values, which was deemed to be a reasonable assumption given that approximately 68% of all days analysed had zero rainfall. Any season in which data were not available for all months within season were removed.

Rainfall measures, including total rainfall, number of rainfall days, and number of rain events were calculated for each season. The distribution of rainfall within each season was calculated in terms of rainfall evenness using Pielou’s formula (Pielou 1966) (note that on days with no rainfall, a small value must be added as this index does not take into consideration ‘0’ values). We determined whether there was any associated trend in each of these values over time using simple linear regression. All statistical analyses were performed using R version 3.5.1 (R Team 2018).

## Results

### Rainfall, pool size and hydroperiod

The duration of rain events was highly variable across both seasons, ranging from as short as 1 day to as long as 8 days (mean = 2.39, SD = 1.72). The interval between rain events was also variable, ranging from 1 to 15 days (mean = 3.58, SD = 2.86). Total rainfall per rain event ranged from 0.4 to 250.60 mm (mean = 22.63, SD = 40.08). Pools used for spawning ranged from 540 to 3,000,000 cm^3^ in volume, and 1.8–16.3 cm in depth.

Pools used for spawning contained water for 49–99% of all days in season 1 and for 43–98% of all days in season 2. However, mean hydroperiods were short because most pools dried repeatedly within each season. Mean hydroperiods in season one ranged from 12 to 126 days, with half of all pools used for spawning possessing mean hydroperiods of less than 28 days. Likewise, mean hydroperiods in season 2 ranged between 6 and 89 days, with more than 90% of pools used for spawning possessing mean hydroperiods of less than 28 days. Hydroperiod length was positively correlated with pool size (*T* = 3.749, *P* = 0.0007) for pools used for spawning.

### Spawning activity

**Fig. 2:**
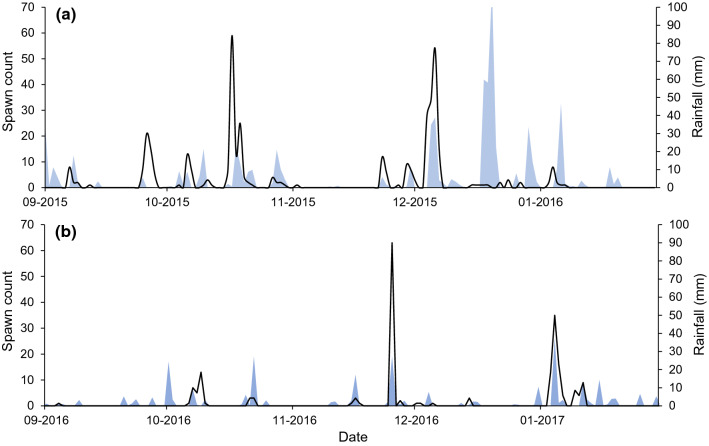
The association between rainfall and spawning activity across two consecutive *Lechriodus fletcheri* breeding seasons in the Watagan Mountains. Total daily rainfall (blue area) has been presented alongside total number of daily oviposited spawn for the **a** 2015/16 and **b** 2016/17 seasons

A combined total of 635 spawns were recorded across the 2015/16 (*n* = 403) and 2016/17 (*n* = 232) breeding seasons (Fig. 2). Spawning occurred in 21 rain events over these two seasons, with the number of spawns laid per event ranging from 1 to 111. When considered across the entire length of the breeding season, a majority of spawning activity was concentrated around periods of rainfall. A majority of spawns were laid during rain events (83%), particularly within the first 3 days from the start of rainfall. The remaining spawns were laid outside of rain events (17%), but within 1–2 days of rainfall. More spawns were laid during periods of higher rainfall (model 1; *Z* = 2.969, *P* = 0.003), with a 1 mm increase in rainfall amount around the oviposition date associated with a 0.24% increase in spawn number. In terms of pool use, spawns were recorded in 34 out of 84 (40%) and 17 out of 71 (24%) pools surveyed in the first and second seasons, respectively. The smaller ephemeral pools surveyed in the study site tended to be avoided, with a 1% increase in size associated with a 33% increase in the mean number of spawns present across the season (model 2; *Z* = 10.381, *P* < 0.0001).

### Clutch success

**Fig. 3:**
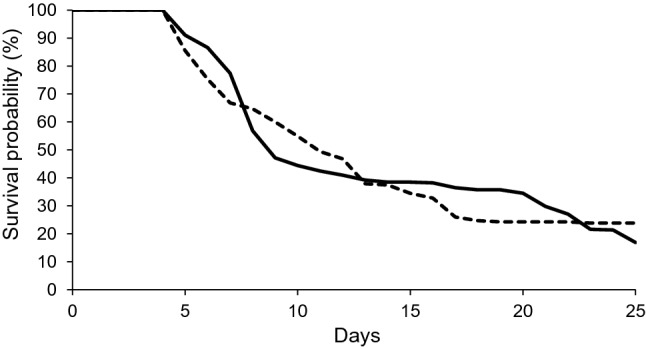
Survival curves depicting the estimated percentage of *Lechriodus fletcheri* spawn with offspring surviving up to 25 days post-oviposition. Spawns were recorded in pools in the Watagan Mountains during the 2015/16 (solid line; *n* = 403) and 2016/17 (dotted line, *n* = 232) breeding seasons

**Fig. 4:**
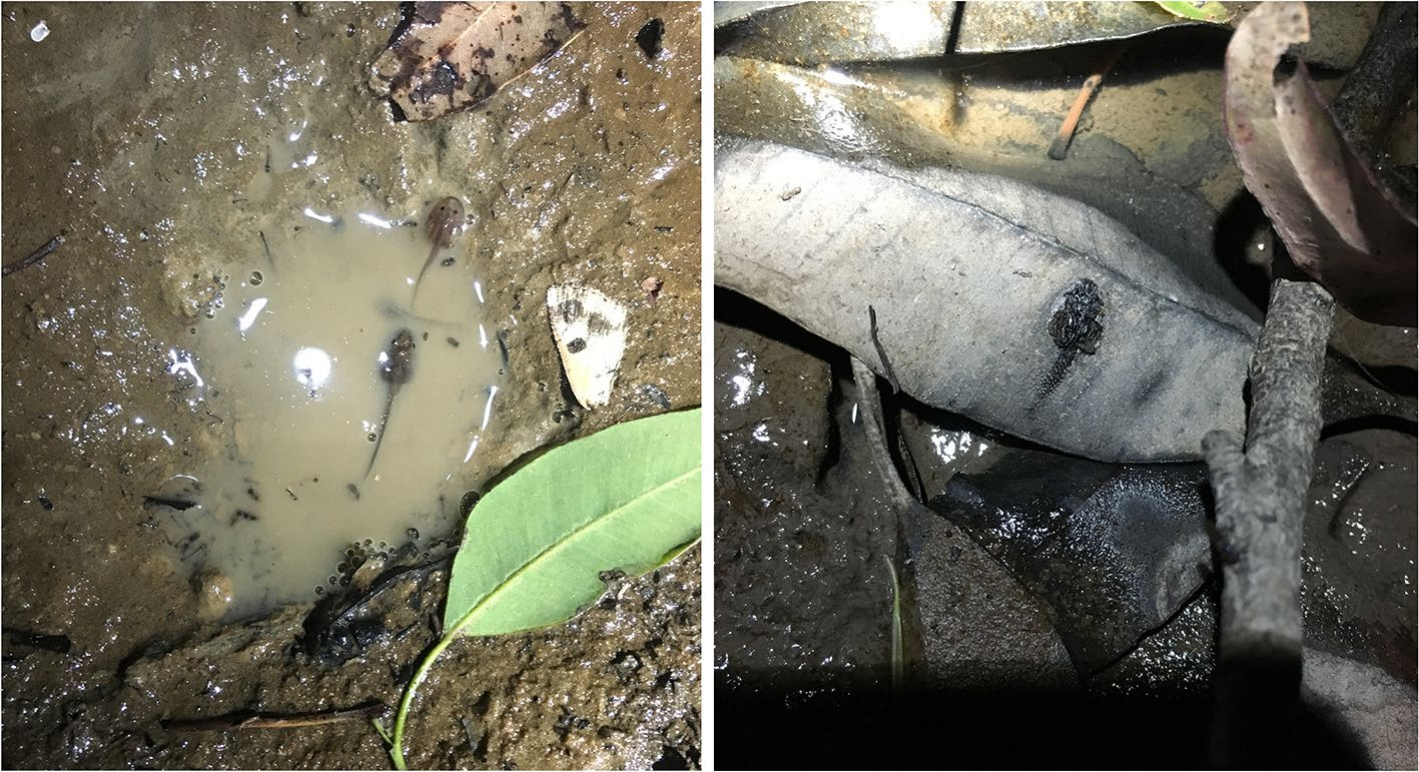
Breeding pools in the Watagan Mountains showing *Lechriodus fletcheri* tadpoles surviving within a small residual volume of water of high turbidity that is close to complete evaporation (left), and decaying tadpole carcass on the surface of a leaf in the sediment of a nearly dry pool (right)

Intense monitoring of spawning events showed that reproductive failure was much more common than success in the ephemeral breeding pools used by *L. fletcheri* under any scenario of tadpole development. Only 32% of spawns gave rise to offspring that survived if the minimum time period to metamorphosis was 17 days; this dropped to 19% if the minimum time period was 25 days (Fig. 3). In the majority of cases, clutch failure was a direct result of pool desiccation prior to the completion of tadpole development (96% in season one, 75% in season two). On many occasions, tadpoles were found in pools that were close to completely drying out, with only small puddles of muddy water remaining (Fig. 4). Tadpoles in this state would quickly succumb to the effects of total pool desiccation, as evidenced by the presence of dried carcasses, often just hours later (Fig. 4).

**Fig. 5:**
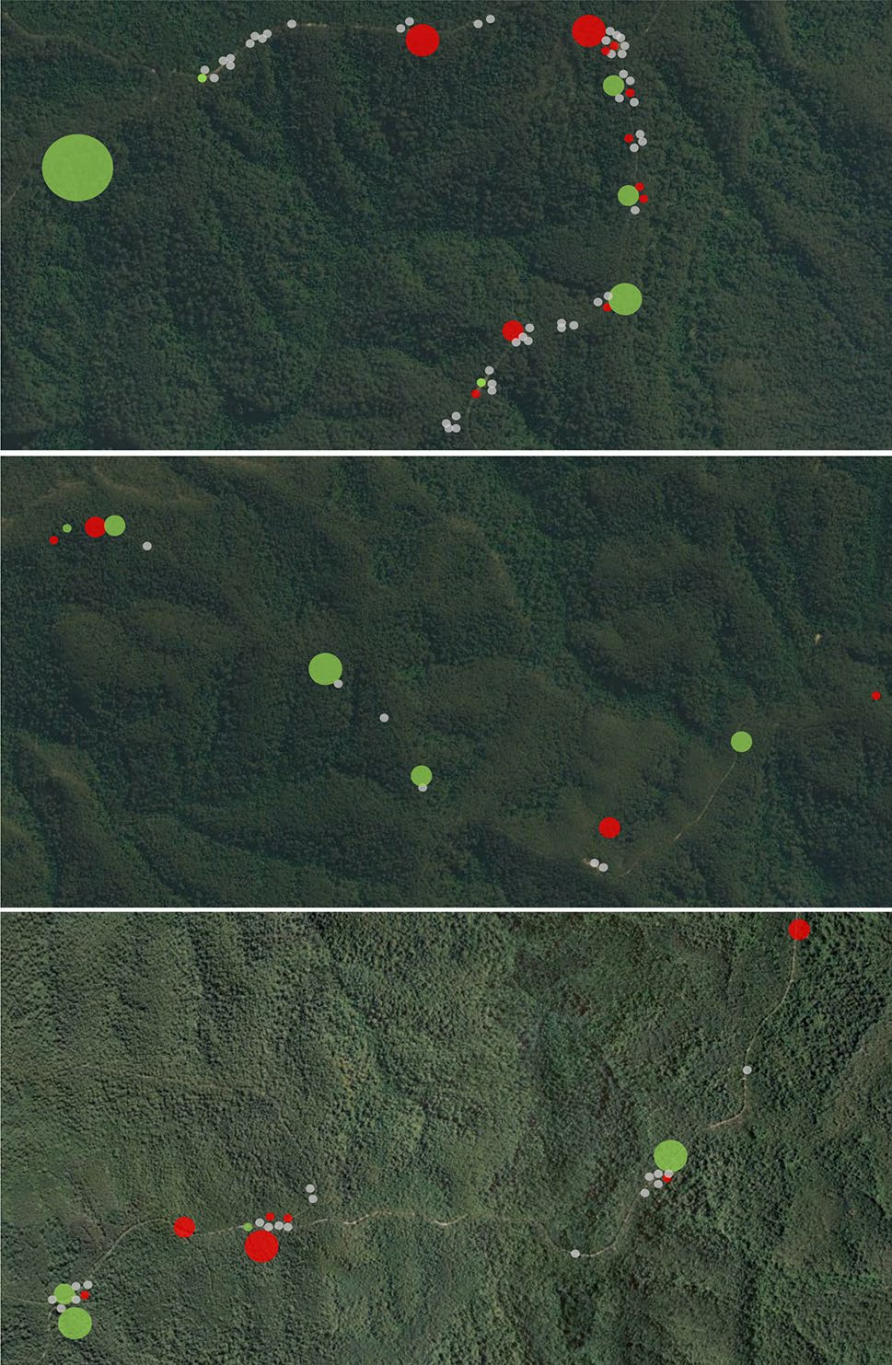
Pools surveyed within the Watagan Mountains across the 2015/16 and 2016/17 *Lechriodus fletcheri* breeding seasons. Each circle represents a single pool, with the size of the circle representing the relative number of spawns that were laid within each across both seasons. The colour of each circle represents the status of the pool including: no spawning occurred (grey), used for spawning and successful metamorphosis occurred at least once (green), and used for spawning but successful metamorphosis was not recorded (red)

Generalised linear modelling indicated that the probability of clutch success was not related to pool size (*Z* = 0.348, *P* = 0.728) or to rainfall quantity during the oviposition period (*Z* =  − 0.709, *P* = 0.479). Cox modelling showed that the risk of offspring mortality declined by 89% with a one unit increase in the evenness of rainfall during the development period after oviposition (*Z* =  − 5.16, *P* < 0.0001), with no significant effect of cumulative total rainfall over development (*Z* = 0.44, *P* = 0.15). In contrast to the generalised linear model, the cox model also showed that clutch success was related to pool size, with the risk of offspring mortality declining by 23% with a one unit increase in the log of pool volume (*Z* =  − 4.14, *P* < 0.0001). We are cautious about the contradictory results for the effect of pool size between the two models. We propose that the true effect of pool size on clutch success can only be accounted for when also considering the effect of rainfall over the entirety of the developmental period, as both have an effect on how long pools remain full of water. This combined effect is not accounted for in the generalised linear model, which does not consider the temporal change in rainfall over time and, thus, could be masking the true effect of pool size on survival (Fig. 5).

In general, adults were frequently unsuccessful at selecting appropriate locations and times for oviposition that would lead to successful metamorphosis. Successful metamorphosis only resulted from eggs oviposited in 11 out of 34 pools (32%) and in 8 out of 11 rain events (73%) surveyed in the first season, and in 5 out of 17 pools (29%) and in 6 out of 10 rain events (60%) surveyed in the subsequent season. Taken together, these data indicate that adult *L. fletcheri* did not have the capacity to select pools or times with a high probability of successful breeding outcomes, nor to learn from repeated failures of breeding in pools with a history of non-viability.

### Historical seasonal rainfall patterns

Over the past nearly 120 years, rainfall has remained highly intermittent within each season, with rain event lengths, dates and intervals remaining highly variable. Taken together, the data indicate that the 2015/16 and 2016/17 breeding seasons were not unusual rainfall years when considered against a period covering most of the last century. Consequently, breeding success and survival outcomes in these seasons are not likely to reflect unusual patterns for reproduction and recruitment.

**Fig. 6:**
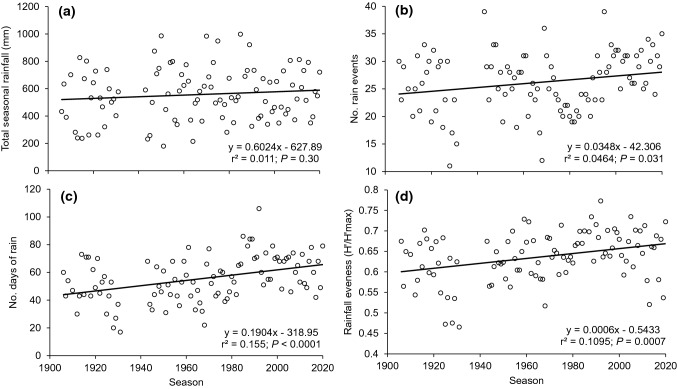
Changes in rainfall patterns across the *Lechriodus fletcheri* breeding season (September–March) from 1906 to 2020. Variables considered include **a** total rainfall (mm), **b** number of rain events, **c** number of days with rainfall above 0.2 mm, and **d** evenness of rainfall per season using Pielou’s formula (Pielou 1966). Rainfall data were collected from the closest weather station (Cooranbong ID: 061,412) located 8–10 km from the study site within the Watagan Mountains

Total seasonal rainfall has not trended with time (*T* = 1.049, *P* = 0.297; Fig. 6A). However, we found a slight increases over time in the number of rain events (*T* = 2.196, *P* = 0.0305; Fig. 6B), number of days of rainfall (*T* = 4.262, *P* < 0.0001; Fig. 6C), and evenness of rainfall within the breeding season (*T* = 3.488, *P* = 0.0007; Fig. 6D).

## Discussion

Offspring survival was low in both breeding seasons, with only a third of *L. fletcheri* spawns giving rise to offspring that reached metamorphosis even when considering the most rapid rate of tadpole development, with total clutch mortality occurring in 100% of spawns laid in several rain events. Clutch failure was primarily due to desiccation as a result of the extremely short hydroperiods of pools, which dried repeatedly and thus often did not contain water for sufficient time for offspring to complete development. Increased probability of clutch success was associated with an increase in the evenness of rainfall during tadpole development, but not with increased rainfall amount, highlighting the limited water holding capacity of pools which require continuous recharging to prevent them from shifting into a dry phase prematurely. Our findings indicate that rainfall unpredictability and intermittency are primary factors generating a landscape that is frequently inhospitable for reproduction in ephemeral pools. Yet, historical data show that both have been constant features of the breeding environment for this species, which has persisted despite offspring survival likely being low each year as a result.

Given the low chances of reproductive success, there should be strong selective pressure for adults to synchronise their spawning with periods that provide suitable conditions for their offspring (Duellman and Trueb 1986). Adaptations that support this include (i) a long, but defined reproductive season over the summer–spring months of the year where climatic conditions of rainfall (but also temperature) are more likely to be favourable (Clulow and Swan 2018), and (ii) a capacity to respond rapidly during the season to adjust the timing of spawning to discrete but unpredictable rain events. Given the rapid rate of pool desiccation, adults are restricted to breeding during rainfall, as most pools are dry and so unavailable for spawning at any other time. Spawning at the start of rain events is thus likely to confer a fitness advantage to offspring by maximising the length of time offspring are in pools before water levels begin to recede after rainfall in that rain event ceases. It is also likely to be a safer time for adults to move towards breeding sites by reducing their own risk of desiccation (Rittenhouse et al. 2009). Further to this, spawning activity was often concentrated around periods of greater rainfall, suggesting that adults are responding to rainfall amounts, not simply the presence or absence of rain, as yet another form of behavioural plasticity in the timing of reproduction. A positive correlation between spawning activity and rainfall has also been found in other amphibian species (Marsh 2000; Goldberg et al. 2006), and is clearly an adaptive response to a variable breeding environment that reduces the risk of offspring mortality due to desiccation. Yet, the response of *L. fletcheri* adults to rainfall amount as a trigger for spawning was not a strong predictor of their offspring’s success.

Pool drying prior to the completion of metamorphosis has been recorded in several species that exploit temporary systems, even in wet years (Newman 1987; Pechmann et al. 1989; Rowe and Dunson 1995; Loman and Claesson 2003), reinforcing that desiccation-induced mortality as a result of short hydroperiods is a critical limiting factor for many amphibians. Given the intermittency of rainfall during the *L. fletcheri* breeding season and consequent unpredictability of the occurrence of follow-up rainfall within the window needed to reach metamorphosis, there is no means by which adults can predict future rainfall based on current conditions encountered at the time of spawning. As such, although spawn may be oviposited under seemingly optimal conditions (e.g. within a particular period of high rainfall), this provides little to no information regarding whether future rainfall will be sufficient to maintain pool hydroperiod. This is in contrast to other amphibians that may better assess desiccation risk in waterbodies that are less ephemeral or reliant on continuous rainfall (Spieler and Linsenmair 1997). Rates of drying of spawning sites used by amphibians generally tend to increase towards the end of the breeding season (Rowe and Dunson 1995), and survival rates as low as 1% have been recorded in species such as *Rana pretisoa* over particularly dry periods (Licht 1974). In contrast, sites used by *L. fletcheri* for spawning oscillate between dry and wet states continuously over the breeding season in a pattern determined by rainfall. As such, survival of progeny in this species may be more closely associated with rainfall intermittency than has previously been recognised for other amphibians.

The long breeding season coupled with the short tadpole phase of *L. fletcheri* are both likely to increase the probability of at least one successful breeding event per year in their highly variable environment. Indeed, there were multiple rain events in both seasons surveyed that gave rise to successful spawning outcomes. An extended season also allows for an increased capacity for adults to participate in multiple reproductive events, which has been shown in both *L. fletcheri* males and females (Gould 2020). This improves adult fitness by allowing them to spread their reproductive potential across multiple rain events within season: a highly advantageous trait in an environment with unreliable rainfall cues.

The rate at which each pool dries after rainfall has ceased is partially dictated by attributes of the pool itself, with those that dry more gradually requiring less follow-up rain after spawning has occurred to replenish water levels as offspring complete development. This could explain the greater spawning activity that occurred in larger pools which possessed longer hydroperiods. Indeed, cox modelling indicated that pool size had a positive effect on survival probability when considered over the length of the developmental period. This suggests that adults can, to some extent, predict hydroperiod by observing pool size. However, even the largest of pools used for spawning dried over the season, with clutch failure detected across all spawning sites irrespective of their size.

Despite the low chances of offspring survival within ephemeral pools, there is apparently strong selection against the use of permanent water bodies by *L. fletcheri* adults, even though permanent ponds were close by and available for use throughout the breeding season within the study site. There are likely to be two selective pressures driving this behaviour: (i) given that sympatric frog species rarely used the ephemeral pools for reproduction, *L. fletcheri* has made the transition to these systems of extreme ephemerality to reduce the exposure of their offspring to competition arising from the greater species richness that generally forms in more permanent systems with longer hydroperiods (Wilbur 1987; Babbitt et al. 2003; Semlitsch et al. 2015), and (ii) it would be expected that exploiting ephemeral systems reduces predation threat as regular drying regimes prevent predator species, particularly fish, from colonising (Skelly 1996; Wellborn et al. 1996). By breeding as soon as pools fill with water, *L. fletcheri* offspring also gain an advantage over those predators that do exploit these systems, which must develop to reach competency to catch and consume prey (however, see Gould et al. 2019). The use of ephemeral pools thus minimises these threats during offspring development, but at the expense of more variable reproductive success and the increased risk of total clutch failure due to desiccation.

In a sister study, we found that reproductively active *L. fletcheri* males often exploit the same pool site between capture events, suggesting that they are remaining at or returning to the same pool for additional bouts of breeding (Gould 2020). This behaviour is paradoxical, as site fidelity should be selected against when site persistence (hydroperiod) is highly variable across the season (Ronce 2007), and it is recognised that dispersal between sites occurs frequently in other amphibian species (Marsh et al. 1999). We found that this behaviour in *L. fletcheri* even occurred in individuals occupying pools that never lasted for sufficient time for offspring to complete development, suggesting that adults are unaware of the failure of any of their previous offspring and their repeated poor choice of breeding location. In a breeding environment where hydroperiod is mainly dictated by rainfall, which is intermittent over the season, and where the duration of breeding sites thus changes unpredictably, it may be more advantageous to remain at the same site and reproduce opportunistically in every possible period of rainfall, rather than several different sites, as a form of temporal rather than spatial bet hedging (Stearns 1977). Other variables are likely to influence pool selection and usage and must be considered, such as the presence/absence of conspecifics within the landscape. Indeed, we have recently shown that *L. fletcheri* avoid spawning in pools with previously hatched conspecific tadpoles already present (Gould et al. 2021a), which we hypothesise is to protect offspring from cannibalism (Gould et al. 2020).

Our findings highlight how species that exploit temporary aquatic sites are faced with the constant threat of total reproductive failure as their offspring race to complete development prior to system dry up. Yet, this dilemma appears to be extreme for *L. fletcheri* offspring, where there are clearly only a very small number of windows of opportunity over the breeding season in which rainfall patterns result in sufficiently long hydroperiods. In a system with unreliable rainfall cues, adults must seek alternative avenues to maximise fitness. This could include the spreading of reproductive potential across multiple breeding sites via clutch-partitioning, or rain events via multiple reproductive episodes, both of which need further investigation for ephemeral pool breeding species. In addition to these adult-derived traits, offspring survival can also be improved by selecting for a rapid developmental rate and developmental plasticity (Roff et al. 2002). Indeed, amphibian species that exploit temporary systems have fast life histories with offspring that can alter the timing of the onset of metamorphosis (e.g. Newman 1989); the limit of which needs to be determined for *L. fletcheri*. While pool drying appears to be the primary driver of reproductive failure in *L. fletcheri*, there are likely to be several causes of offspring mortality, such as predation (e.g. cannibalism; Gould et al. 2020) that must also be considered.

It is likely that *L. fletcheri* requires large networks of potential breeding sites and possibly metapopulations for long-term persistence, as indicated for other amphibians (Gómez-Rodríguez et al. 2009), given the high chance that many, if not all, sites will become sub-optimal for offspring survival at various times over the breeding season. The suitability of breeding sites may be impacted by changes in hydroperiods as a result of climate change-related shifts in weather patterns and increases in rainfall variability (MacCracken et al. 2003; Thomas et al. 2004). However, historical rainfall data indicate that regional conditions are apparently becoming more suitable for offspring survival over time, with the potential benefits of climate change recorded in other amphibians (McCaffery and Maxell 2010). Nonetheless, it will be important to continue monitoring rates of clutch success for species that exclusively use temporary aquatic sites for reproduction, as they may be less resilient to environmental change than expected.

## Supplementary Information

Below is the link to the electronic supplementary material.

Supplementary file1 (XLSX 117 KB)

## Data Availability

Datasets used during the current study are available from the corresponding author on reasonable request.

## References

[CR1] Abney CR, Sterling WB, Ashley D, Andrew B, David RC (2019). Early spring and early vanishing wetlands as harbingers of the future? The climate change trap for ephemeral pond-breeding frogs. Northwest Sci.

[CR2] Anstis M (2017). Tadpoles and frogs of Australia.

[CR3] Babbitt KJ, Baber MJ, Tarr TL (2003). Patterns of larval amphibian distribution along a wetland hydroperiod gradient. Can J Zool.

[CR4] Batzer D, Boix D (2016). Invertebrates in freshwater wetlands.

[CR5] Black JH (1976). Environmental fluctuations in central Oklahoma temporary ponds. Proc Okla Acad Sci.

[CR6] Blair WF (1960). A breeding population of the Mexican toad (*Bufo valliceps*) in relation to its environment. Ecology.

[CR7] Brooks RT (2009). Potential impacts of global climate change on the hydrology and ecology of ephemeral freshwater systems of the forests of the northeastern United States. Clim Change.

[CR8] Brunner P, Cook PG, Simmons CT (2011). Disconnected surface water and groundwater: from theory to practice. Groundwater.

[CR9] Chang A, Olmstead W, Johanson J, Yamashita G (1974). The sealing mechanism of wastewater ponds. Jwater Pollut Control Fed.

[CR10] Clulow S, Swan M (2018). A complete guide to frogs of Australia.

[CR11] Colburn EA, Weeks SC, Reed SK, Calhoun AJK, deMaynadier PG (2008). Diversity and ecology of vernal pool invertebrates. Science and conservation of vernal pools in northeastern North America.

[CR12] Cox DR (1972). Regression models and life-tables. J R Stat Soc Series B Methodol.

[CR13] Denver RJ, Mirhadi N, Phillips M (1998). Adaptive plasticity in amphibian metamorphosis: response of *Scaphiopus hammondiitadpoles* to habitat desiccation. Ecology.

[CR14] Duellman WE, Lizana M (1994). Biology of a sit-and-wait predator, the leptodactylid frog *Ceratophrys cornuta*. Herpetologica.

[CR15] Duellman W, Trueb L (1986). Biology of amphibians.

[CR16] Feder ME, Burggren WW (1992). Environmental physiology of the amphibians.

[CR17] Fredlund D, Xing A, Huang S (1994). Predicting the permeability function for unsaturated soils using the soil-water characteristic curve. Can Geotech J.

[CR18] Furness AI, Lee K, Reznick DN (2015). Adaptation in a variable environment: phenotypic plasticity and bet-hedging during egg diapause and hatching in an annual killifish. Evolution.

[CR19] Goldberg F, Quinzio S, Vaira M (2006). Oviposition-site selection by the toad *Melanophryniscus rubriventris* in an unpredictable environment in Argentina. Can J Zool.

[CR20] Gómez-Rodríguez C, Díaz-Paniagua C, Serrano L, Florencio M, Portheault A (2009). Mediterranean temporary ponds as amphibian breeding habitats: the importance of preserving pond networks. Aquat Ecol.

[CR21] Gosner KL (1960). A simplified table for staging anuran embryos and larvae with notes on identification. Herpetologica.

[CR22] Gould J, Jose WV, Clulow J, Clulow S (2019). Diving beetle offspring oviposited in amphibian spawn prey on the tadpoles upon hatching. Entomol Sci.

[CR23] Gould J, Clulow J, Clulow S (2020). Food, not friend: tadpoles of the sandpaper frog (*Lechriodus fletcheri*) cannibalise conspecific eggs as a food resource in ephemeral pools. Ethology.

[CR24] Gould J, Clulow J, Rippon P, Doody JS, Clulow S (2021). Complex trade-offs in oviposition site selection in a cannibalistic frog. Anim Behav.

[CR25] Gould J, Valdez J, Clulow J, Clulow S (2021). Left high and dry: froth nesting allows eggs of the anuran amphibian to complete embryogenesis in the absence of free-standing water. Ichthyol Herpetol.

[CR26] Gould J (2020) Risky business in Ephemeral Waters: the reproductive ecology of the sandpaper frog, *Lechriodus fletcheri*. PhD dissertation, School of Environmental and Life Sciences, University of Newcastle, Newcastle, New South Wales, Australia

[CR27] Heyer WR, Mcdiarmid RW, Weigmann DL (1975). Tadpoles, predation and pond habitats in the tropics. Biotropica.

[CR28] Licht LE (1974). Survival of embryos, tadpoles, and adults of the frogs *Rana aurora aurora* and *Rana pretiosa pretiosa* sympatric in southwestern British Columbia. Can J Zool.

[CR29] Loman J, Claesson D (2003). Plastic response to pond drying in tadpoles *Rana temporaria*: tests of cost models. Evol Ecol.

[CR30] Maccracken MC, Barron EJ, Easterling DR, Felzer BS, Karl TR (2003). Climate change scenarios for the US national assessment. Bull Am Meteorol Soc.

[CR31] Marsh DM (2000). Variable responses to rainfall by breeding tungara frogs. Copeia.

[CR32] Marsh DM, Fegraus EH, Harrison S (1999). Effects of breeding pond isolation on the spatial and temporal dynamics of pond use by the tungara frog, *Physalaemus pustulosus*. J Anim Ecol.

[CR33] McCaffery RM, Maxell BA (2010). Decreased winter severity increases viability of a montane frog population. PNAS.

[CR34] Nager RG, Van Noordwijk AJ (1995). Proximate and ultimate aspects of phenotypic plasticity in timing of great tit breeding in a heterogeneous environment. Am Nat.

[CR35] Newman R (1987). Effects of density and predation on *Scaphiopus couchi* tadpoles in desert ponds. Oecologia.

[CR36] Newman RA (1989). Developmental plasticity of *Scaphiopus couchii* tadpoles in an unpredictable environment. Ecology.

[CR37] Pechmann JH, Scott DE, Gibbons JW, Semlitsch RD (1989). Influence of wetland hydroperiod on diversity and abundance of metamorphosing juvenile amphibians. Wetl Ecol Manag.

[CR38] Pielou EC (1966). The measurement of diversity in different types of biological collections. J Theor Biol.

[CR39] R Team (2018). R: a language and environment for statistical computing.

[CR40] Rittenhouse TA, Semlitsch RD, Thompson Iii FR (2009). Survival costs associated with wood frog breeding migrations: effects of timber harvest and drought. Ecology.

[CR41] Roff DA, Mostowy S, Fairbairn DJ (2002). The evolution of trade-offs: testing predictions on response to selection and environmental variation. Evolution.

[CR42] Ronce O (2007). How does it feel to be like a rolling stone? Ten questions about dispersal evolution. Annu Rev Ecol Evol Syst.

[CR43] Rowe CL, Dunson WA (1995). Impacts of hydroperiod on growth and survival of larval amphibians in temporary ponds of central Pennsylvania, USA. Oecologia.

[CR44] Saward-Arav D, Sadeh A, Mangel M, Templeton AR, Blaustein L (2016). Oviposition responses of two mosquito species to pool size and predator presence: varying trade-offs between desiccation and predation risks. Isr J Ecol Evol.

[CR45] Schneider DW, Frost TM (1996). Habitat duration and community structure in temporary ponds. J North Am Benthol Soc.

[CR46] Semlitsch RD, Peterman WE, Anderson TL, Drake DL, Ousterhout BH (2015). Intermediate pond sizes contain the highest density, richness, and diversity of pond-breeding amphibians. PLoS ONE.

[CR47] Shoop CR (1974). Yearly variation in larval survival of *Ambystoma maculatum*. Ecology.

[CR48] Skelly DK (1996). Pond drying, predators, and the distribution of *Pseudacris* tadpoles. Copeia.

[CR49] Skelly DK, Werner EE, Cortwright SA (1999). Long-term distributional dynamics of a Michigan amphibian assemblage. Ecology.

[CR50] Spieler M, Linsenmair K (1997). Choice of optimal oviposition sites by *Hoplobatrachus occipitalis* (Anura: Ranidae) in an unpredictable and patchy environment. Oecologia.

[CR51] Stearns SC (1977). The evolution of life history traits: a critique of the theory and a review of the data. Annu Rev Ecol Syst.

[CR52] Thomas CD, Cameron A, Green RE, Bakkenes M, Beaumont LJ, Collingham YC, Erasmus BF, De Siqueira MF, Grainger A, Hannah L (2004). Extinction risk from climate change. Nature.

[CR53] Wellborn GA, Skelly DK, Werner EE (1996). Mechanisms creating community structure across a freshwater habitat gradient. Annu Rev Ecol Syst.

[CR54] Werner EE, Mcpeek MA (1994). Direct and indirect effects of predators on two anuran species along an environmental gradient. Ecology.

[CR55] Wilbur HM (1980). Complex life cycles. Ann Rev Ecol Syst.

[CR56] Wilbur HM (1987). Regulation of structure in complex systems: experimental temporary pond communities. Ecology.

[CR57] Williams DD (2006). The biology of temporary waters.

[CR58] Zacharias I, Dimitriou E, Dekker A, Dorsman E (2007). Overview of temporary ponds in the Mediterranean region: threats, management and conservation issues. J Environ Biol.

